# Forecasting solar energetic particles using multi-source data from solar flares, CMEs, and radio bursts with machine learning approaches

**DOI:** 10.1038/s41598-025-92207-1

**Published:** 2025-03-19

**Authors:** Mohammed AbuBakr Ali, Ali G. A. Abdelkawy, Abdelrazek M. K. Shaltout, M. M. Beheary

**Affiliations:** 1 Department of Space Environment, National Authority for Remote Sensing and Space Science (NARSS), Cairo, 11769 Egypt; 2 Faculty of Science, Department of Astronomy and Meteorology, Al-Azhar University, Cairo, 11884 Egypt

**Keywords:** Solar energetic particle, Solar flare, Radio burst, Machine learning, Forecasting, Astronomy and planetary science, Astronomy and astrophysics, Space physics

## Abstract

This study presents a consistent method to the inherently imbalanced problem of predicting solar energetic particle (SEP) events, using a variety of datasets that include solar flares, coronal mass ejections (CMEs), and radio bursts. We applied several machine learning (ML) methods, including Random Forests (RF), Decision Trees (dtree), and Support Vector Machines (SVM) with both linear (linSVM) and nonlinear (svm) kernels. To assess model performance, we used standard metrics such as Probability of Detection (POD), False Alarm Rate (FAR), True Skill Statistic (TSS), and Heidke Skill Score (HSS). Our results show that the RF model consistently outperforms the other algorithms across datasets containing flares, CMEs, and radio bursts. For the sweep frequency dataset, RF achieved a POD of $0.85 (\pm 0.08)$, a FAR of $0.30 (\pm 0.05)$, a TSS of $0.78 (\pm 0.07)$,and a HSS of $0.71 (\pm 0.03$). For the fixed-frequency dataset, RF produced a POD of $0.76 (\pm 0.12)$, a FAR of $0.31 (\pm 0.08)$, a TSS of $0.71 (\pm 0.11)$ ,and a HSS of $0.67 (\pm 0.06$). Key features for SEP prediction include CME linear speed and angular width across both datasets. For sweep frequency, flare intensity and integral soft X-ray (SXR) flux are crucial, while for fixed frequency, the rise time and duration of radio bursts at 1415 MHz are significant.

## Introduction

Solar Energetic Particle (SEP) events, which are significant occurrences in space weather, occur due to the acceleration of large numbers of electrons, protons, and heavy-ion nuclei. This acceleration takes place at two distinct locations: solar flares and interplanetary shocks generated by CMEs. Forbush^1^ performed the first observations of SEP events in the middle of the 1940s, utilising ground-based ionisation chambers and neutron monitors to find energies ranging from kiloelectron volts (KeV) up to gigaelectron volts (GeV)^2^.

There are three approaches to predict SEPs. Empirical models are based on identifying connections and interactions in observational data that might be associated with fundamental physical mechanisms. Physics-based modeling utilizes our current comprehension of particle acceleration and transport phenomena on the Sun and interplanetary space to replicate these events and predict the characteristics of subsequent SEPs occurrences. Machine Learning (ML) techniques are currently being investigated to produce a new class of SEP models that provide quick forecasts with an increased accuracy^3^. ML encompasses a group of approaches to extract knowledge and correlations from the data for a variety of applications.These techniques have been used to identify patterns in datasets by performing several tasks such as regression, classification, and clustering.

The field of SEP prediction has utilized various ML algorithms. For instance,Núñez et al.^4^ employed an interpretable decision tree model to predict SEP events with energies exceeding 10 MeV using solar flare and radio burst data. The study utilized data spanning from 1997 to 2014, achieving a Probability of Detection (POD) of 0.702, indicating its ability to accurately identify actual SEP events. The reported False Alarm Rate (FAR) was 0.402, reflecting the rate of false alarms generated by the model. The average anticipation time provided was 9 hours and 52 minutes, offering a substantial lead time for forecasting SEP events.

Aminalragia-Giamini et al.^5^ employed a Neural Networks (NN) approach to forecast the likelihood of SEP events. They utilized soft X-ray (SXR) data from solar flares associated with SEPs measured at 1 AU, spanning from 1988 to 2013. The neural network demonstrated remarkable accuracy, with a prediction rate exceeding 0.85 for correctly identifying SEP occurrences and over 0.92 for accurately predicting instances without SEP events. The National Oceanic and Atmospheric Administration (NOAA) employs a scale that categorizes space weather into five levels: minor (S1), moderate (S2), strong (S3), severe (S4), and extreme (S5)^6^. The Empirical model for Solar Proton Event Real Time Alert (ESPERTA) forecasting tool for $\ge$S1 events achieved a POD of 0.63, a FAR of 0.38, and a warning time (WT) of 4.8 hours (median) and 0.4 hours (minimum). The WT is calculated as the difference between the time the alert is issued (10 minutes after the $\ge$M2 SXR peak) and the onset of the SEP event (end of the third consecutive 5-minute interval with high proton flux). Laurenza et al.^7^ modified the ESPERTA model to predict S2 events for the period 1995-2014, based on predictions made at the time of the S1 threshold crossing. The modified model achieved a POD of 0.75 (41 out of 55 events correctly predicted), a FAR of 0.24 (13 out of 54 false alarms), and median and minimum warning times of 1.7 and 0.2 hours, respectively.

Stumpo et al.^8^ re-analyzed the ESPERTA system using a logistic regression model and employed three input parameters: the heliolongitude of the flare, the SXR fluence, and the fluence of radio signals at 1 MHz. Their study achieved a POD of 0.83 and a FAR of 0.39. Lavasa et al.^9^ evaluated the efficiency of various machine learning (ML) techniques-including logistic regression (LR), Support Vector Machine (SVM), NN using a fully connected multi-layer perceptron (MLP), Random Forest (RF), Decision Trees (DTs), Extremely Randomized Trees (XT), and Extreme Gradient Boosting (XGB)-for predicting SEP features using datasets related to solar flares and CMEs. They reported a POD of $0.76 \pm 0.06$, a FAR of $0.34 \pm 0.10$, a True Skill Statistic (TSS) of $0.75 \pm 0.05$, and a Heidke Skill Score (HSS) of $0.69 \pm 0.04$.

Kasapis et al.^10^ conducted a study using Space-Weather Michelson Doppler Imager (MDI) Active Region Patches (SMARPs) and regression models, specifically logistic and ridge regressions, to forecast SEP events resulting from active regions producing flares. Their findings indicated an accuracy (ACC) of $0.72 \pm 0.12$ and a competitive lead time of $55.3 \pm 28.6$ minutes for predicting SEP events.

Núñez et al.^11^ developed an empirical model called UMASEP (University of Málaga Solar Particle Event Predictor) to forecast SEPs with energies exceeding 10 MeV. The UMASEP model adopts a dual-model approach. The first model predicts well-connected SEP events using an improved lag-correlation algorithm that analyzes soft X-ray (SXR) emissions and differential proton fluxes, estimating the Sun-Earth magnetic connectivity empirically. The second model focuses on forecasting poorly connected events by studying the evolution of differential proton fluxes. Notably, the UMASEP model demonstrated its highest performance during solar cycle 24 (2008-2019), achieving a POD of 0.911 (41 out of 45 events correctly predicted), a FAR of 0.128 (6 out of 47 false alarms), and an average WT of 2 hours and 46 minutes.

Various additional approaches have been proposed to analyze solar flare and radio data to predict the incidence of SEP events with energies exceeding 10 MeV. These alternative methods and techniques offer different perspectives and insights into forecasting SEP events, contributing to the advancement in the field of space weather prediction^7,12,13^.

The objective of this research is to forecast the occurrence of SEP events with particle energies exceeding 10 MeV, specifically focusing on events with a particle flux threshold of 10 pfu, by utilizing data from solar flares, CMEs and radio bursts. The research is centered on the application of machine learning methods to forecast >10 MeV SEP events. This was achieved by utilizing source data files from NOAA Space Weather Prediction Center (NOAA/SWPC) and observations captured by the Large Angle and Spectrometric Coronagraph (LASCO) instrument aboard the Solar and Heliospheric Observatory (SOHO). The main aim of the developed model is to improve the detection probability, reduce the false alarm ratio, and enhance the accuracy of the average warning time (AWT). To estimate the anticipation time, the model utilizes observational data from flare and radio burst measurements, along with CME data, which together provide insight into the early stages of physical processes, such as magnetic reconnection, shock formation, and CME propagation, that can lead to solar energetic particle events^14^. In this study, we conducted a comparative analysis of our forecasting results with three existing models: ESPERTA^7^, the University of Malaga predictor from Solar Data (UMASOD)^4^, and the Lavasa model^9^. Our research focused on four machine learning models, namely Decision Trees, Random Forests, linear/nonlinear kernel SVM to predict the incidence of >10 MeV SEP events. For this analysis, the data utilized spanned from 1997 to 2022 and consisted of solar flare, CME and radio burst data (Tables 1, 2, 3).

## Results and discussion

**Fig. 1:**
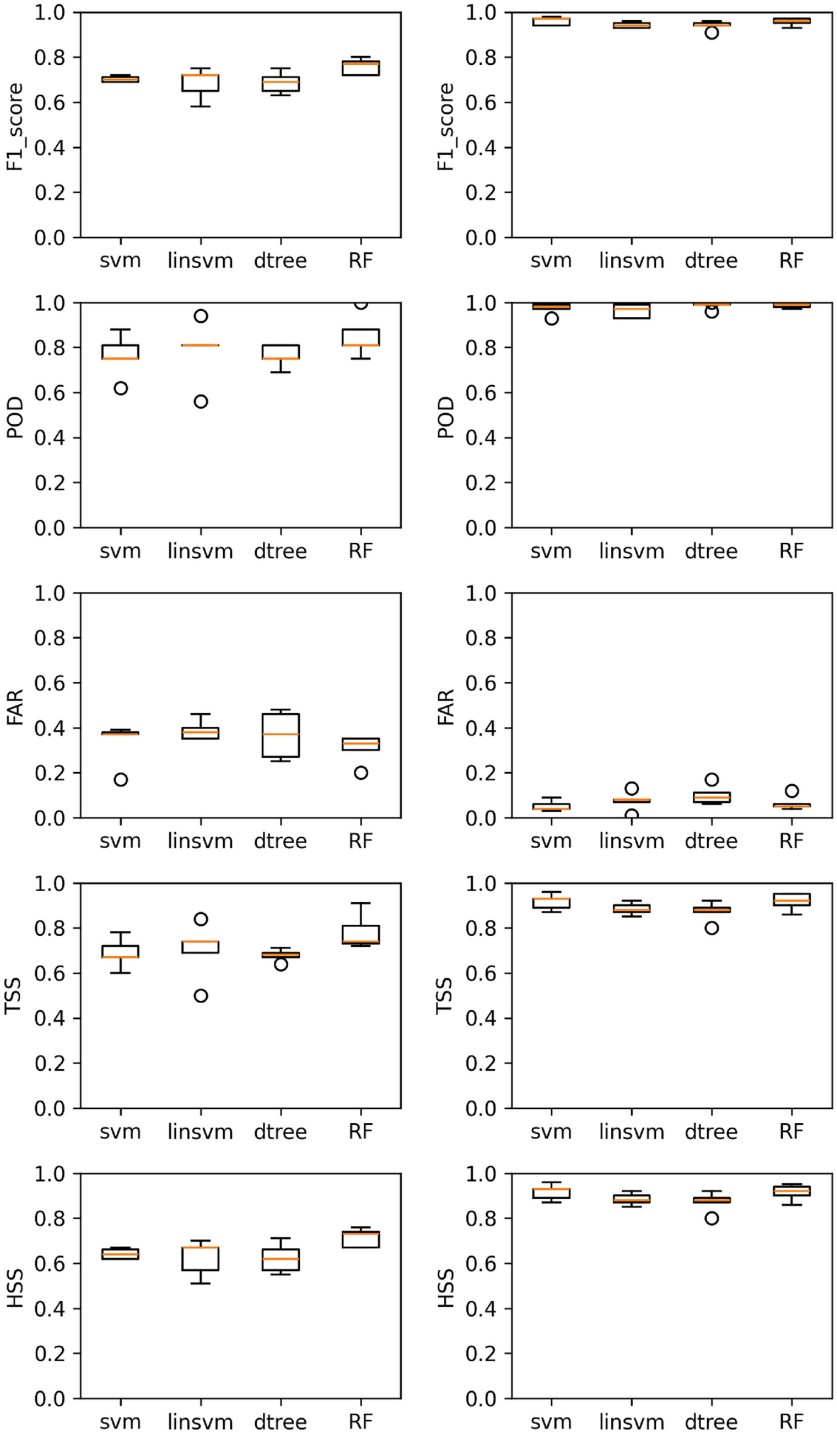
Models’ performance plots, from top to bottom: F1-scores, POD, FAR, TSS and HSS, on the sweep frequency dataset. We display the results obtained by the Imbalanced (left panel) and Balanced using SMOTE (right panel) evaluation settings under F1 optimization. The scores are constructed for each of the four models employed, which are printed on the x-axis. The circle indicates outliers.

**Fig. 2:**
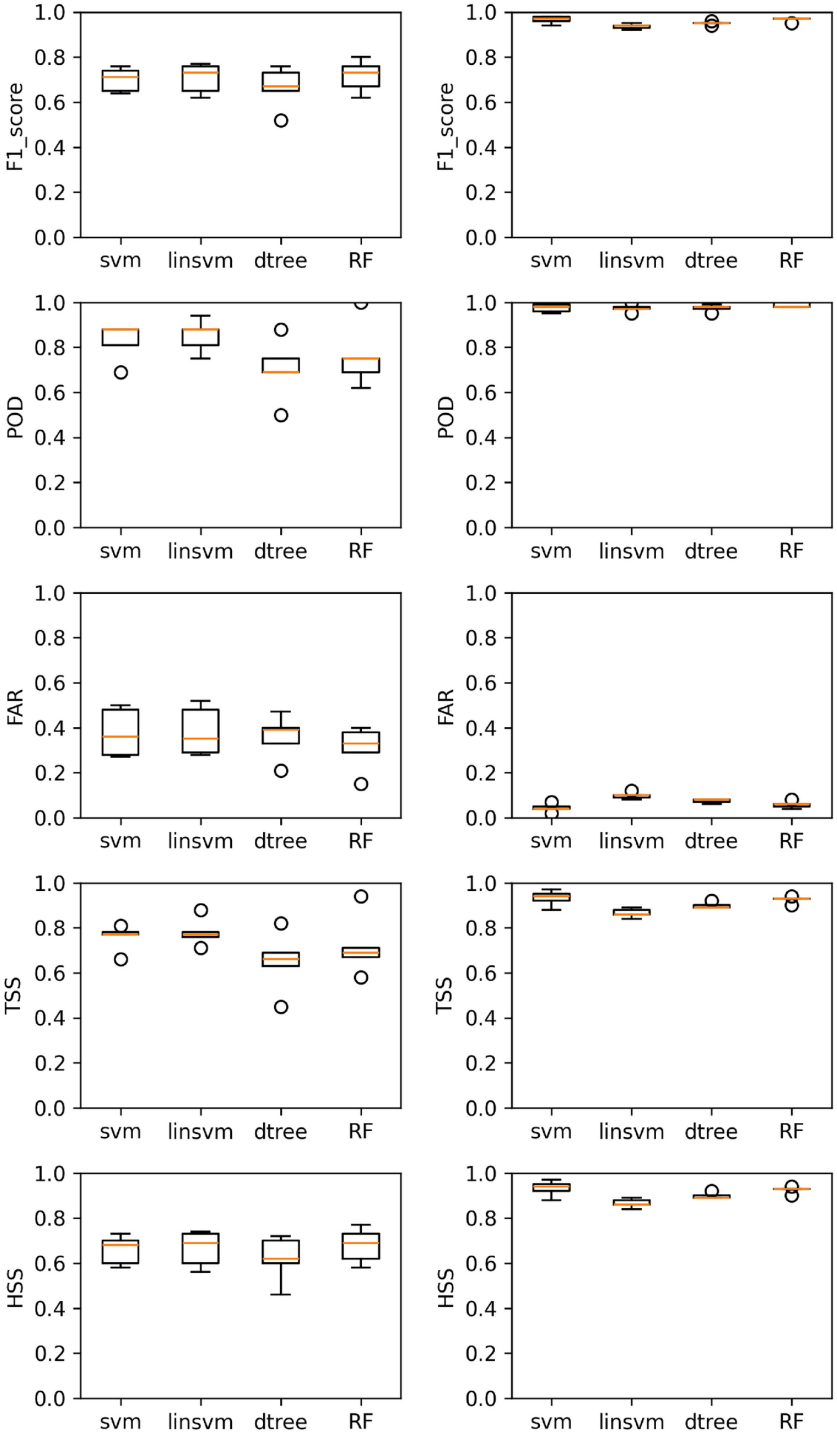
Models’ performance plots, from top to bottom: F1-scores, POD, FAR, TSS and HSS, on the fixed frequency dataset. We display results obtained by the Imbalanced (left panel) and Balanced (right panel) evaluation settings under F1 optimization. The scores are constructed for each of the four models employed, which are printed on the x-axis. The circle indicates outliers.

**Fig. 3:**
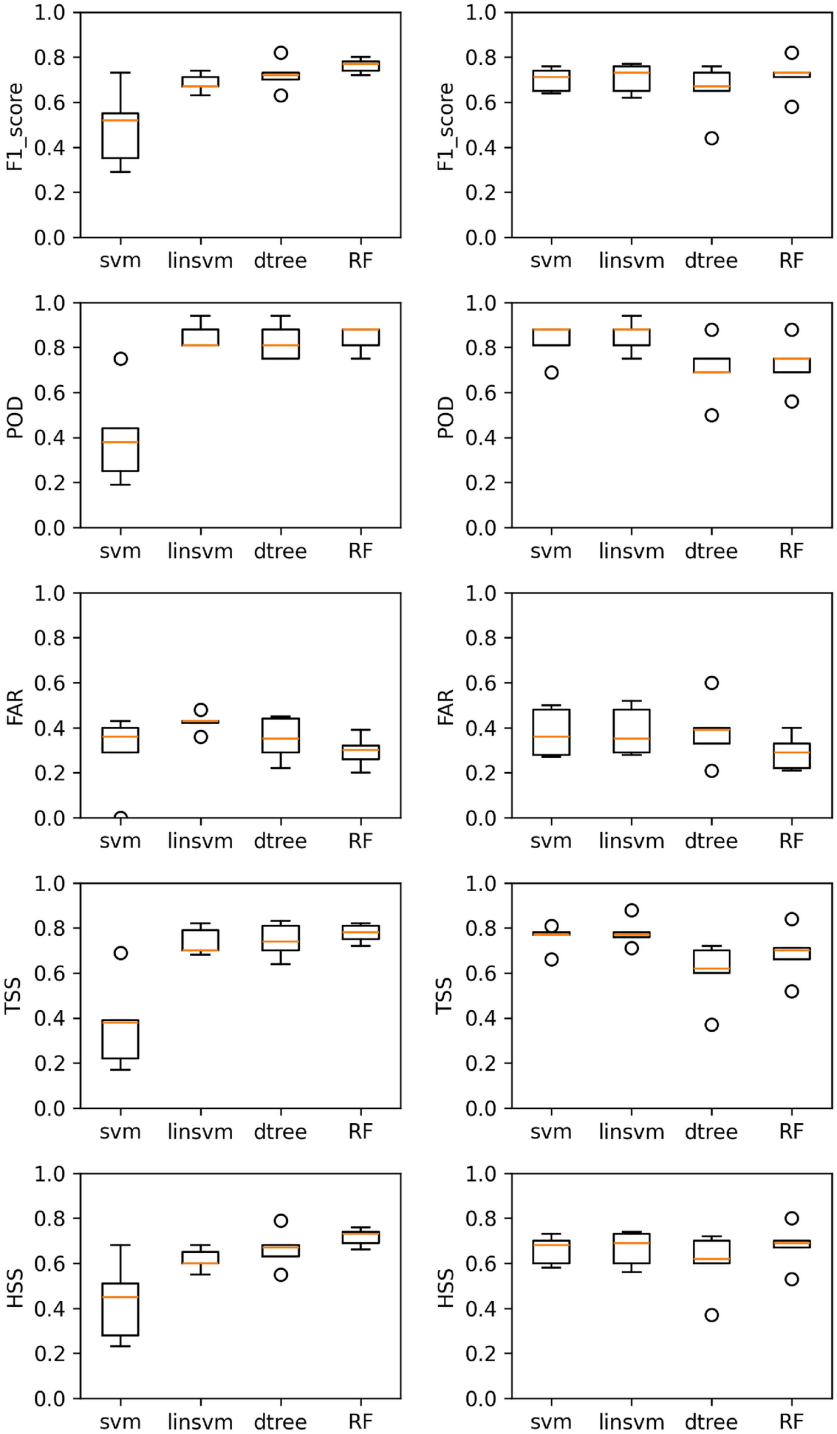
Performance plots of the models, from top to bottom: F1-scores, POD, FAR, TSS, and HSS, for the two datasets using the Hybrid method. Results are shown for the Sweep Frequency dataset (left panel) and the Fixed Frequency dataset (right panel) under F1 optimization. The scores are presented for each of the four models, labeled on the x-axis. Outliers are indicated by circles.

In this study, we focus to predict SEPs by integrating multi-source data, including CMEs, Solar Flares, and radio bursts. We used machine learning (ML) models alongside traditional statistical techniques to predict Solar Energetic Particle (SEP) events. Specifically, we apply the following models: Decision Tree (dtree), Random Forests (RF), Support Vector Machines (svm), and Linear Support Vector Machines (linSVM). Additionally, we implement a nested cross-validation pipeline. As described earlier, we have tested three evaluation settings-Imbalanced, Balanced, and Hybrid-using two datasets (Sweep Frequency and Fixed Frequency). To evaluate our models, we use probabilistic metrics such as POD, FAR, TSS, HSS, and F1-Score. The results are presented from table 4 to table 16, showing the metrics for the Imbalanced, Balanced, and Hybrid settings. These numbers are averaged across the folds of a nested cross-validation scheme, using unseen test partitions. Figures 1, 2, and 3, along with Tables 4, 5, and 6, depict the performance of four models under the scenarios of Imbalanced, Balanced, and Hybrid data settings across two datasets.

For the balanced case, as shown in Table 5 and Figs. 1 and 2, all models achieve the following performance metrics: POD > 96%, FAR < 10%, F1-score > 93%, and HSS and TSS > 86%. The SVM model stands out as the superior choice for both the Sweep Frequency and Fixed Frequency datasets.Similarly, for the hybrid case, as shown in Table 6 and Fig. 3, all models achieve POD >= 40%, FAR < 42%, F1-score >= 48%, HSS >=43% , and TSS >= 37%. Again, the RF model demonstrates superior performance across both datasets.

**Table 1:** Solar radio burst classifications with its characteristics.

Type	Characteristics	Duration	Frequency range	Associated phenomena
I	Short bursts with limited bandwidth, are often seen in large numbers alongside a continuous signal	Single burst: $\sim$ 1 second storm: hours–days	80–200 MHz	Active regions, flares, eruptive prominences
II	Bursts exhibit low-frequency drift, usually accompanied by a varying second harmonic	3–30 min	20–150 MHz	Proton emission, flares, and magnetohydrodynamic shockwaves
III	Rapidly drifting bursts that can occur alone, in groups, or storms, often with a continuous underlying signal. They may also have a second harmonic	Single burst: 1–3 s group: 1–5 min storm: minutes–hours	10 kHz–1GHz	Active regions, flares
IV	Broadband continuum with intricate structure, referred to as stationary type IV	Hours–days	20 MHz–2GHz	Flares, proton emission
V	Smooth and short-lived continuity, observed after some type III explosions. It never occurs independently	1–3 min	10–200 MHz	Same as type III bursts

**Table 2:** The difference in minutes between the end of fixed frequency radio burst and the start of the SEP event.

Date	Associated flare	Location	Radio burst at 245 MHZ	Radio burst at 410 MHZ	Radio burst at 606 MHZ	Radio burst at 1415 MHZ	Radio burst at 2695 MHZ	Radio burst at 4995 MHZ	Radio burst at 8800 MHZ	Radio burst at 15,400 MHZ
4-Nov-1997	X2.1	S14W33	131	131	140	146	146	146	148	143
6-Nov-1997	X9.4	S18W63	45	49	36	42	46	50	56	40
2-May-1998	X1.1	S15W15	$-45$	$-43$	$-53$	$-53$	$-53$	$-53$	$-53$	$-53$
6-May-1998	X2.7	S11W65	39	18	17	17	24	24	25	21
24-Aug-1998	X1.0	N30E07	53	79	80	79	53	53	53	53
23-Sep-1998	M7.1	N18E09	2423	2423	2423	2427	2423	2415	2429	2447
30-Sep-1998	M2.8	N23W81	120	70	120	70	74	74	70	70
20-Jan-1999	M5.2	N27E90		–	–	3763	3695	3695	3833	3695
3-May-1999	M4.4	N15E32	3599	3597	3599	3586	3590	3565	3575	3569
4-Jun-1999	M3.9	N17W69	129	138	141	141	137	129	135	135
6-Jun-2000	X2.3	N20E18	1345	1288	1288	1288	1288	1288	1284	1288
10-Jun-2000	M5.2	N22W38	58	56	52	52	52	41	43	57
14-Jul-2000	X5.7	N22W07	$-47$	$-47$	$-47$	$-28$	$-71$	$-74$	$-72$	$-71$
22-Jul-2000	M3.7	N14W56	82	118	82	99	99	98	98	98
16-Oct-2000	M2.5	N04W90	–	–	235	233	235	236	–	–
8-Nov-2000	M7.4	N10W77	45	49	24	$-37$	$-51$	$-51$	$-51$	$-22$
24-Nov-2000	X2.0	N20W05	614	614	610	613	613	608	600	604
29-Mar-2001	X1.7	N14W12	349	359	402	406	–	350	402	316
2-Apr-2001	X20	N18W82	86	99	95	17	19	13	19	24
10-Apr-2001	X2.3	S23W09	67	67	103	112	103	104	103	119
15-Apr-2001	X14.4	S20W85	$-14$	$-64$	$-71$	$-71$	$-71$	$-71$	$-71$	$-71$
26-Apr-2001	M7.8	N17W31	2447	2375	2373	2447	2353	2373	2373	2373
24-Sep-2001	X2.6	S16E23	–	$-41$	$-41$	33	24	$-69$	$-68$	$-41$
1-Oct-2001	M9.1	S22W91	–	–	–	391	390	392	390	405
19-Oct-2001	X1.6	N15W29	320	320	320	320	320	320	320	320
22-Oct-2001	X1.2	S18E16	62	62	62	62	62	61	61	61
4-Nov-2001	X1.0	N06W18	$-38$	$-9$	$-4$	$-22$	6	$-16$	$-20$	$-20$
17-Nov-2001	M2.8	S13E42	3332	3336	3283	3341	3217	3219	3256	3240
22-Nov-2001	M9.9	S15W34	–	–	–	–	–	–	–	–
26-Dec-2001	M7.14	N08W54	–	$-38$	$-39$	$-43$	$-50$	$-55$	$-61$	$-37$
28-Dec-2001	X3.4	S26E90	550	547	543	505	496	485	517	485
20-Feb-2002	M5.1	N12W72	94	95	96	79	94	94	78	77

Date	Associated flare	Location	Radio burst at 245 MHZ	Radio burst at 410 MHZ	Radio burst at 606 MHZ	Radio burst at 1415 MHZ	Radio burst at 2695 MHZ	Radio burst at 4995 MHZ	Radio burst at 8800 MHZ	**Radio burst at 15,400 MHZ**
15-Mar-2002	M2.2	S08W03	1994	1994	1997	2000	1997	1996	1996	2037
17-Apr-2002	M2.6	S14W34	337	289	289	285	285	293	318	297
21-Apr-2002	X1.5	S14W84	-41	-105	-78	-105	-105	-103	-103	-103
15-Jul-2002	X3.0	N19W01	1169	1081	1165	1162	–	–	1204	1194
14-Aug-2002	M2.3	N09W54	377	–	385	381	372	352	374	374
22-Aug-2002	M5.4	S07W62	–	167	166	167	162	158	154	152
24-Aug-2002	X3.1	S08W90	12	34	37	-6	-27	-33	-39	-24
9-Nov-2002	M4.6	S12W29	335	355	355	353	352	345	345	331
28-May-2003	X3.6	S07W17	1316	1317	1331	1334	1372	1356	1346	1359
31-May-2003	M9.3	S07W65	108	109	142	126	125	110	109	113
17-Jun-2003	M6.8	S08E61	1276	1276	1281	1288	1288	1292	1294	1298
26-Oct-2003	X1.2	N02W38	7	7	7	5	-36	-36	-36	-36
28-Oct-2003	X17.2	S16E08	-173	-173	-173	-166	-164	-153	-161	-161
20-Nov-2003	M5.8	N02W17	5	5	5	5	5	5	5	5
12-Sep-2004	M4.8	N04E42	2604	2601	2580	2556	2548	2539	2554	2557
7-Nov-2004	X2.0	N09W17	95	82	101	96	97	111	120	126
15-Jan-2005	X2.6	N15W05	1484	1503	1564	1567	1548	1546	1553	1541
13-May-2005	M8.0	N12E11	708	721	705	709	728	709	703	703
16-Jun-2005	M4.0	N09W87	–	108	108	-120	104	92	95	106
13-Jul-2005	M5.0	N10W80	743	744	753	728	722	703	701	700
27-Jul-2005	M3.7	N11E90	1079	1074	–	–	–	–	1055	1050
22-Aug-2005	M5.6	S12W60	213	213	213	213	213	213	213	213
7-Sep-2005	X17.0	S06E89	454	452	442	430	425	433	433	433
5-Dec-2006	X9.0	S07E79	1760	1736	1751	1741	1739	1738	1737	1730
13-Dec-2006	X3.4	S05W23	–	–	-16	-41	-36	-29	-29	-33
7-Mar-2011	M3.7	N24W59	233	234	–	227	227	317	317	–
7-Jun-2011	M2.5	S21W64	96	115	–	95	95	95	97	98
4-Aug-2011	M9.3	N15W49	132	132	–	155	154	153	153	153
9-Aug-2011	X6.9	N17W83	36	37	–	39	39	38	38	38
22-Sep-2011	X1.4	N11E74	–	–	–	2149	2140	2052	2053	2033
23-Jan-2012	M8.7	N28W36	82	77	–	64	64	60	62	65

Date	Associated flare	Location	Radio burst at 245 MHZ	Radio burst at 410 MHZ	Radio burst at 606 MHZ	Radio burst at 1415 MHZ	Radio burst at 2695 MHZ	Radio burst at 4995 MHZ	Radio burst at 8800 MHZ	Radio burst at 15,400 MHZ
27-Jan-2012	X1.7	N27W71	42	45	–	16	11	-8	15	-9
7-Mar-2012	X5.4	N17E15	187	186	–	181	180	278	168	–
13-Mar-2012	M7.9	N18W62	23	–	–	5	–	6	6	–
17-May-2012	M5.1	N12W89	12	20	–	6	18	15	21	26
6-Jul-2012	X1.1	S18W50	293	293	–	291	291	289	277	–
12-Jul-2012	X1.4	S16W09	-95	63	–	89	90	81	11	20
11-Apr-2013	M6.5	N09E12	116	130	–	164	217	219	212	225
15-May-2013	X1.2	N11E51	699	703	–	699	695	694	692	691
22-May-2013	M5.0	N15W70	67	68	–	42	42	42	42	49
21-Jun-2013	M2.9	S16E66	3886	3883	–	3885	3884	3904	–	–
7-Jan-2014	X1.2	S15W11	558	585	–	581	581	604	600	602
20-Feb-2014	M3.0	S15W67	59	64	–	54	54	54	55	63
25-Feb-2014	X4.9	S12E82	781	782	–	772	768	765	761	757
4-Sep-2017	M5.5	S08W16	–	639	–	–	–	–	637	–
20-Jan-2022	M5.5	N08W68	50	50	–	50	50	56	71	108
28-Mar-2022	M4.0	N13W06	53	55	–	91	–	113	113	114
2-Apr-2022	M4.3	N14W65	-201	–	–	–	-200	–	–	–

**Table 3:** The percentage of radio burst events that take place after the SEP.

Type	Number of radio burst	After SEP	Percentage (%)
Radio burst at 245 MHZ	69	8	11.59
Radio burst at 410 MHZ	71	8	11.26
Radio burst at 606 MHZ	53	9	16.98
Radio burst at 1415 MHZ	76	11	14.47
Radio burst at 2695 MHZ	73	11	15.07
Radio burst at 4995 MHZ	75	13	17.33
Radio burst at 8800 MHZ	76	12	15.78
Radio burst at 15400 MHZ	71	13	18.30

**Table 4:** The models’ performance when dealing with imbalanced data across both sweep and fixed frequencies files.

Dataset	Models	F1_score	POD	FAR	TSS	HSS
Sweep frequency (imbalance)	dtree	0.68(±0.04)	0.76(±0.04)	0.36(±0.09)	0.67(±0.02)	0.62(±0.05)
	RF	0.75(±0.03)	0.85(±0.08)	0.30(±0.05)	0.78(±0.07)	0.71(±0.03)
	svm	0.70(±0.01)	0.76(±0.08)	0.33(±0.08)	0.68(±0.05)	0.64(±0.02)
	linsvm	0.68(±0.06)	0.78(±0.12)	0.38(±0.04)	0.70(±0.11)	0.62(±0.07)
Fixed frequency (imbalance)	dtree	0.66(±0.08)	0.70(±0.12)	0.36(±0.08)	0.65(±0.11)	0.62(±0.09)
	RF	0.71(±0.06)	0.76(±0.12)	0.31(±0.08)	0.71(±0.11)	0.67(±0.06)
	svm	0.7(±0.04)	0.82(±0.07)	0.37(±0.09)	0.75(±0.05)	0.65(±0.05)
	linsvm	0.70(±0.06)	0.85(±0.06)	0.38(±0.09)	0.78(±0.05)	0.66(±0.07)

**Table 5:** The models’ performance in the context of balanced data.

Dataset	Models	F1_score	POD	FAR	TSS	HSS
Sweep frequency (balance)	dtree	0.94(±0.01)	0.98(±0.01)	0.1(±0.03)	0.87(±0.03)	0.87(±0.03)
	RF	0.95(±0.01)	0.98(±0.01)	0.06(±0.02)	0.91(±0.03)	0.91(±0.03)
	svm	0.96(±0.01)	0.97(±0.02)	0.05(±0.02)	0.91(±0.03)	0.91(±0.03)
	linsvm	0.94(±0.01)	0.96(±0.02)	0.07(±0.03)	0.88(±0.02)	0.88(±0.02)
Fixed frequency (balance)	dtree	0.95(±0.006)	0.97(±0.01)	0.07(±0.008)	0.89(±0.01)	0.89(±0.01)
	RF	0.96(±0.008)	0.98(±0.009)	0.05(±0.01)	0.92(±0.01)	0.92(±0.01)
	svm	0.96(±0.01)	0.97(±0.01)	0.04(±0.01)	0.93(±0.03)	0.93(±0.03)
	linsvm	0.93(±0.01)	0.97(±0.01)	0.09(±0.01)	0.86(±0.01)	0.86(±0.01)

**Table 6:** The models’ performance in the context of balanced data.

Dataset	Models	F1_score	POD	FAR	TSS	HSS
Sweep frequency (Hybrid)	dtree	0.72(±0.06)	0.82(±0.07)	0.35(±0.08)	0.74(±0.07)	0.66(±0.07)
	RF	0.76(±0.02)	0.84(±0.05)	0.29(±0.06)	0.77(±0.03)	0.71(±0.03)
	svm	0.48(±0.15)	0.40(±0.19)	0.29(±0.15)	0.37(±0.18)	0.43(±0.16)
	linsvm	0.68(±0.03)	0.85(±0.05)	0.42(±0.03)	0.73(±0.05)	0.61(±0.04)
Fixed frequency (Hybrid)	dtree	0.64(±0.11)	0.70(±0.12)	0.38(±0.12)	0.64(±0.13)	0.60(±0.12)
	RF	0.71(±0.07)	0.72(±0.10)	0.29(±0.07)	0.68(±0.10)	0.67(±0.08)
	svm	0.7(±0.04)	0.82(±0.07)	0.37(±0.09)	0.75(±0.05)	0.65(±0.05)
	linsvm	0.70(±0.06)	0.85(±0.06)	0.38(±0.09)	0.78(±0.05)	0.66(±0.07)

In the Imbalanced evaluation setting, the performance of the classification algorithms varies across both the Sweep Frequency and Fixed Frequency datasets. The RF model stands out as the most consistent performer, achieving high scores across key metrics such as F1-score, POD, FAR, TSS, and HSS, with low variability across both datasets. Specifically, for Sweep Frequency data, RF achieves an F1-score of 0.75 (±0.03), POD of 0.85 (±0.08), FAR of 0.30 (±0.05), TSS of 0.78 (±0.07), and HSS of 0.71 (±0.03). For Fixed Frequency data, RF delivers an F1-score of 0.71 (±0.06), POD of 0.76 (±0.12), FAR of 0.31 (±0.08), TSS of 0.71 (±0.11), and HSS of 0.67 (±0.06). The linsvm model also performs well, with an F1-score of 0.70 (±0.06), POD of 0.85 (±0.06), FAR of 0.38 (±0.09), TSS of 0.78 (±0.05), and HSS of 0.66 (±0.07) for Fixed Frequency data. For Sweep Frequency data, linsvm achieves an F1-score of 0.68 (±0.06), POD of 0.78 (±0.12), FAR of 0.38 (±0.04), TSS of 0.70 (±0.11), and HSS of 0.62 (±0.07). However, its slightly higher FAR compared to RF suggests a trade-off between correctly identifying SEP events and minimizing false alarms. The svm model provides a balanced performance, achieving an F1-score of 0.70 (±0.01), POD of 0.76 (±0.08), FAR of 0.33 (±0.08), TSS of 0.68 (±0.05), and HSS of 0.64 (±0.02) for Sweep Frequency data. For Fixed Frequency data, svm achieves an F1-score of 0.70 (±0.04), POD of 0.82 (±0.07), FAR of 0.37 (±0.09), TSS of 0.75 (±0.05), and HSS of 0.65 (±0.05). The dtree model, while offering reasonable performance, lags behind with lower F1-scores and higher FAR values. For Sweep Frequency data, dtree achieves an F1-score of 0.68 (±0.04), POD of 0.76 (±0.04), FAR of 0.36 (±0.09), TSS of 0.67 (±0.02), and HSS of 0.62 (±0.05). For Fixed Frequency data, dtree delivers an F1-score of 0.66 (±0.08), POD of 0.70 (±0.12), FAR of 0.36 (±0.08), TSS of 0.65 (±0.11), and HSS of 0.62 (±0.09).These results suggest that RF models strike the best balance between identifying SEP events (high POD) and minimizing false positives (low FAR), making them the top choice for SEP prediction in this setting. On the other hand, linsvm and svm models are better suited for scenarios where the focus is on maximizing the detection of SEP events, even at the cost of slightly higher FAR.

As shown in Table 7, Random Forest (RF) models deliver strong performance across the Sweep Frequency dataset. On average, the training scores are high, with a POD of 84%, FAR of 26%, and F1-score of 78%. The validation scores are slightly lower but remain consistent, averaging a POD of 81%, FAR of 23%, and F1-score of 77%. The test scores are also closely aligned, with a POD of 85%, FAR of 30%, and F1-score of 75%. This consistency indicates that RF models handle the complexity of the data well while maintaining strong generalization capabilities.For the Fixed Frequency dataset, RF models perform similarly, achieving average training scores of 82% for POD, 27% for FAR, and 77% for F1-score. The validation scores remain close, with a POD of 76%, FAR of 28%, and F1-score of 73%. Although the test scores show slightly more variation, they are still robust, with averages of 76% for POD, 31% for FAR, and 71% for F1-score.Overall, RF models demonstrate reliable performance across both datasets, with minimal differences between training, validation, and test scores. This consistency confirms that there is no evidence of overfitting in these models.

**Table 7:** The performance of the RF model on the training set, validation set, and test set for both sweep and fixed frequencies data in cases of imbalance data.

Dataset		Train set			Validate set			Test set		
		POD	FAR	F1	POD	FAR	F1	POD	FAR	F1
Sweep frequency (imbalance)	Fold1	0.87	0.26	0.79	0.82	0.26	0.77	0.81	0.35	0.72
	Fold2	0.95	0.21	0.85	0.87	0.28	0.78	0.75	0.2	0.77
	Fold3	0.84	0.33	0.74	0.84	0.26	0.77	0.88	0.3	0.78
	Fold4	0.82	0.26	0.77	0.81	0.19	0.79	1	0.33	0.8
	Fold5	0.74	0.22	0.75	0.71	0.18	0.76	0.81	0.35	0.72
	Avg	0.84	0.26	0.78	0.81	0.23	0.77	0.85	0.30	0.75
	Std	0.06	0.03	0.03	0.05	0.04	0.01	0.08	0.05	0.03
Fixed frequency (imbalance)	Fold1	0.82	0.30	0.75	0.79	0.33	0.72	0.75	0.4	0.67
	Fold2	0.84	0.28	0.77	0.78	0.29	0.73	0.69	0.15	0.76
	Fold3	0.80	0.22	0.79	0.73	0.26	0.73	0.75	0.29	0.73
	Fold4	0.78	0.28	0.74	0.74	0.31	0.71	1	0.33	0.8
	Fold5	0.83	0.26	0.78	0.76	0.23	0.75	0.62	0.38	0.62
	Avg	0.82	0.27	0.77	0.76	0.28	0.73	0.76	0.31	0.71
	Std	0.02	0.02	0.01	0.02	0.03	0.01	0.12	0.08	0.06

**Table 8:** Comparison of different models results.

Model	Period		POD	FAR	AWT
Our model	1997–2022	The sweep frequency file	85 %	30 %	4 h 14 min
		The fixed-frequency file	76 %	31%	4 h 36 min
Our model	1997–2014	The sweep frequency file	80 %	26 %	5 h 4 min
		The fixed-frequency file	80 %	28%	4 h 47 min
Lavasa model	1997 –2013		76 %	34 %	–
UMASOD	1997–2014		70.2 %	40.2%	9h 52 min
ESPERTA	1995–2014		62 %	39 %	9 h
ESPERTA	1995–2005		63 %	42 %	9 h
UMASEP-10	1997–2014		91.1%	12.8%	2 h 46 min

**Fig. 4:**
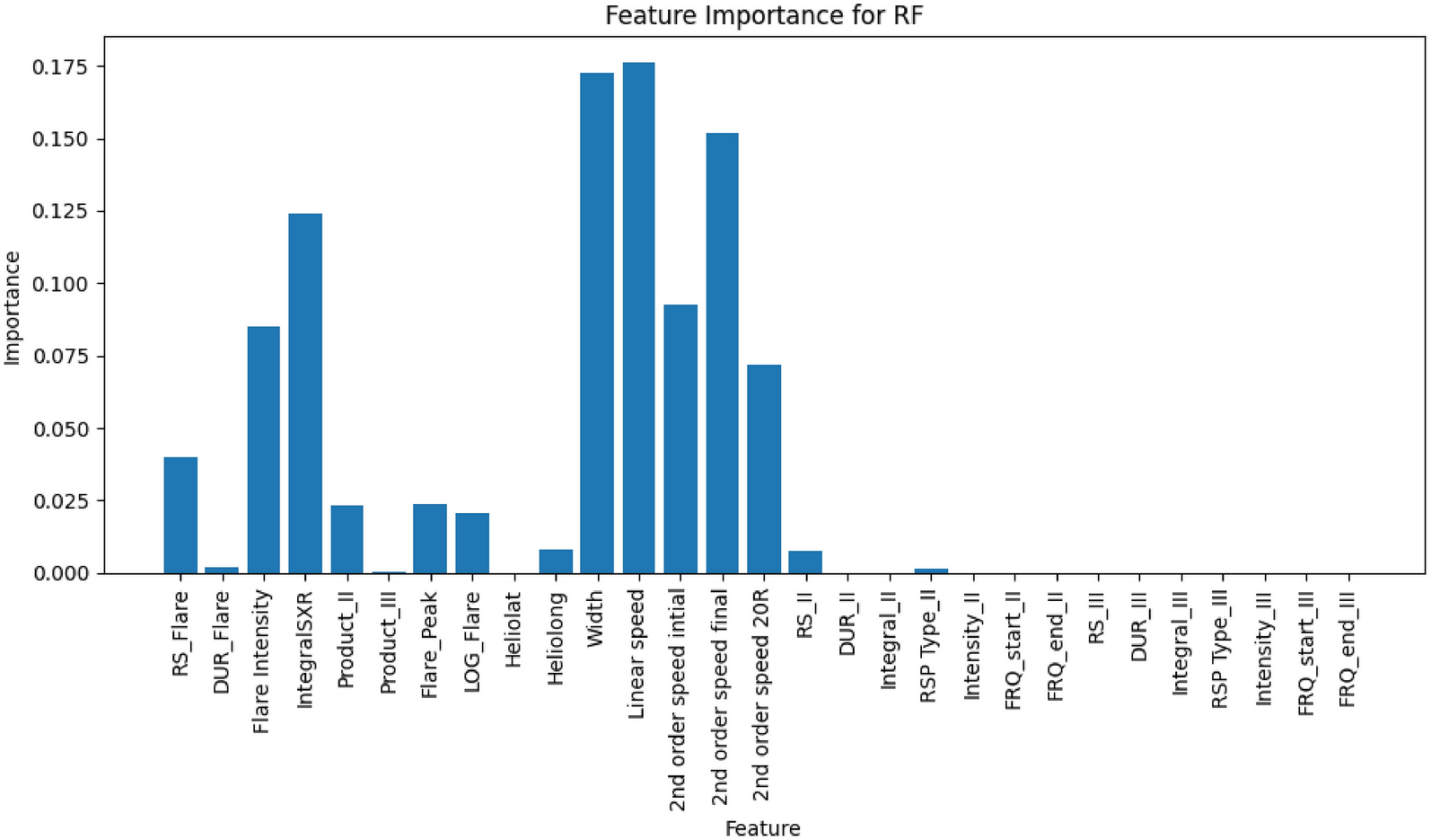
The feature importance of the RF model for the sweep frequency data. The x-axis lists the features used in the model, including solar flare characteristics such as solar flare intensity, integral SXR, as well as CME characteristics like linear speed and angular width. The y-axis represents the importance score of each feature, indicating its contribution to the model’s predictions. This figure demonstrates that features such as linear speed, and angular width play significant roles in the RF model’s performance.

**Fig. 5:**
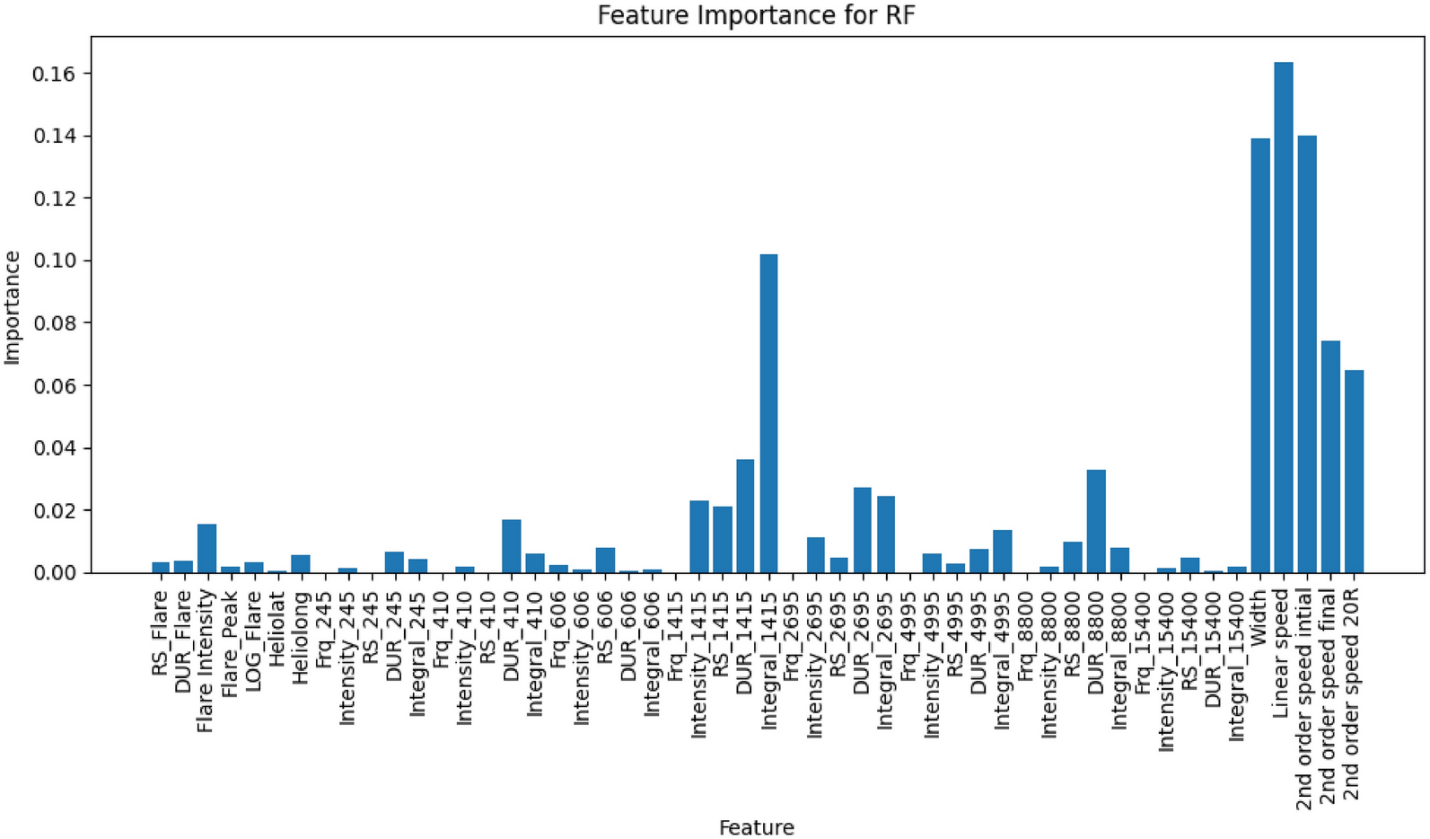
Feature importance scores for the RF model applied to the fixed frequency dataset. The x-axis lists the features used in the model, including solar flare characteristics, CME characteristics, and fixed frequency radio burst characteristics. The y-axis displays the importance score of each feature, reflecting its relative impact on the model’s predictions. Notably, linear speed, angular width, and second order speed of CME emerge as the most significant features, contributing the most to the model’s predictive power.

Figures 4 and 5 show the feature importance for the RF model applied to the sweep frequency and fixed frequency datasets, respectively.For the sweep frequency dataset (Fig. 4), the RF model is primarily driven by CME-related features, such as angular width, linear speed, and second-order speed (initial, final, and at 20 R$_{\odot }$). These features exhibit the highest importance, reflecting their strong relationship with the SEP predictions. Additionally, flare-related features, including flare intensity and integral soft X-ray (SXR), play a significant role, suggesting that they also contribute meaningfully to the model’s performance. Moderately important features include RS_Flare (flare rise time), Product_II, Flare_Peak, Heliolong (heliographic longitude), and RS_II (rise time of Type II radio bursts). These variables, while not as influential as CME and flare characteristics, still add valuable information to the prediction process.Similarly, for the fixed frequency dataset (Fig. 5), CME-related features remain the most significant predictors, with linear speed and second-order speed final contributing the most to the model’s output. Fixed-frequency radio burst features at 1415 MHz, 2695 MHz, and 8800 MHz-including burst intensity, rise time, duration, and integral-also play a prominent role. Among these, the intensity at 1415 MHz stands out as particularly important. Flare intensity and related variables maintain moderate importance, aligning with observations from the sweep frequency dataset. Furthermore, the intensity, integral, and duration of bursts at 2695 MHz influence the model’s performance to a lesser degree than CME and 1415 MHz features.In summary, the results highlight the dominance of CME characteristics and fixed-frequency radio burst features in the RF model’s predictions. Flare-related features serve as secondary predictors. This analysis underscores the importance of prioritizing high-impact features to optimize the model’s predictive performance. From the analysis of feature importance of the two datasets (sweep and fixed frequency datasets), we found that the most significant predictors of SEP events prediction are CME-related features. The significance features of the angular width and linear speed data underscores the key physical role that CMEs play in accelerating solar energetic particles. Cases of faster and wider CME features are more likely to drive large SEP events, and they are associated with stronger shock waves that accelerate particles to high energies. These features play a crucial role in the predictive accuracy of the ML models. The important features in the sweep frequency dataset are flare intensity, flare rise time (RS_Flare) and CME. The flare intensity is linked to the release of high- energy electromagnetic radiation, which accelerates particles contributing to SEP events. RS_Flare offers valuable insights into the initial stages of particle acceleration. In the case of the Fixed Frequency dataset, the characteristics of radio bursts at 1415 MHz serve as strong predictors for SEP, which are associated with electron acceleration and shock formation.

To estimate the AWT (Average Warning Time) accurately, we replicated real-time processes. This involved incorporating a 30-minute delay for SWPC to gather solar event data and 6 hours delay for SOHO/LASCO to gather CME data. The CDAW CME manually Catalog which is needed additional time to be available online rather than automatically generated archives like CACTUS catalog. In fact, the CDAW catalog includes additional parameters such as the 2nd-Order Speed (Initial and Final), and 2nd-Order Speed at 20 Rsun, which was used in the our ML models. Additionally, we updated the modified event list file to align with the end time of the most delayed solar event, such as a radio burst or flare^4^. Based on the sweep frequency file, we have computed the AWT by considering the most delayed event, where these events are solar flare,CME, and radio bursts type (II, III). In the period from 1997 to 2022, the AWT was found to be 4 hours and 14 minutes. For the fixed frequency file, we examined the end time of each event of fixed frequency radio burst and selected the most delayed among them. In the period from 1997 to 2022, the computed AWT was 4 hours and 36 minutes. Our models predict SEPs associated with strong solar flares of class M2.0 or greater. The model is limited to cases where all relevant phenomena (solar flare, CME, and radio bursts) occur and the corresponding data are available for prediction.

Several models have been developed using solar flare and CME data, or solar flare and radio burst data, to predict SEP events. These models differ in terms of input features, time spans, and methodologies. In this study, we applied also the models over the time span of 1997-2014.Table 8 presents the outcomes of our model alongside those of ESPERTA^7,13^, UMASOD^4,15^ and^9^. UMASEP (University of Malaga Solar Particle Event Predictor) correlates solar data with near-earth particle data^15^. The UMASOD model, proposed by Núñez et al.^4^, predicts SEP events using solar flare and Type II and III radio burst features. It considers flares of class $\ge$M2.0 and uses data from 1997 to 2014. UMASOD achieved a POD of 0.70, a FAR of 0.40, and an AWT of 9 hours 52 minutes. We compared our results for the same time span with this model. For the sweep frequency dataset, our results showed a POD of 0.67 and FAR of 0.17 for the dtree model, a POD of 0.76 and FAR of 0.22 for the svm, a POD of 0.78 and FAR of 0.35 for the linSVM, and a POD of 0.80 and FAR of 0.26 for the RF model. For the fixed frequency dataset, dtree yielded a POD of 0.77 and FAR of 0.30, svm achieved a POD of 0.78 and FAR of 0.35, linSVM resulted in a POD of 0.84 and FAR of 0.32, and RF attained a POD of 0.80 and FAR of 0.28.In terms of AWT, our model demonstrated an AWT of 5 hours 4 minutes for the sweep frequency dataset and 4 hours 47 minutes for the fixed frequency dataset. These results highlight that our models surpass UMASOD’s performance during the same period. All models achieved a lower FAR than UMASOD, and three out of four achieved a higher POD. The superior performance of our models can be attributed to the inclusion of CME parameters, which led to higher POD, lower FAR, and significantly reduced AWT.

We also compared our results at time span(1997-2014) with the ESPERTA model, which uses input data such as flare location, flare size, and evidence of particle acceleration/escape parameterized by flare longitude, time-integrated soft X-ray intensity, and time-integrated intensity of type III radio emission at $\sim$ 1 MHz, considering $\ge$M2.0 class flares , covering the period from 1995 to 2005. The ESPERTA model had a POD of (0.62), a FAR of (0.39), and an AWT of 55 minutes. Our results for four models outperformed ESPERTA with higher POD and lower FAR, indicating that our model has superior predictive performance . Our models, by incorporating CME features, outperform previous models like UMASOD and ESPERTA in terms of both POD and FAR. This improvement highlights the importance of accounting for CME dynamics in SEP forecasting, as they significantly enhance the accuracy of predictions. The reduced AWT in our models further confirms the relevance of CME characteristics in predicting SEP events with shorter warning times. Lavasa et al.^9^ trained their model on highly imbalanced data, including flares of class $\ge$C1.0 , where the positive-to-negative class ratio was approximately 0.03 at time span 1997 to 2013 .this model achevied a POD (0.76±0.06) and FAR (0.34±0.10).Our models, using a $\ge$M2.0 flare threshold and incorporating radio burst data, achieved higher performance over the 1997-2014 time span. However, our data and cases are different from those used by Lavasa et al., and therefore, our results are not directly comparable to this model.

## Summary and conclusion

In this study, we employed machine learning techniques to develop predictive models for > 10 MeV SEP events associated with solar flares $\ge$M2.0 class over a significant period from 1997 to 2022.Our approach involved leveraging multi-source data encompassing flares, CMEs, and radio bursts for training models of RF, dtree, and SVM algorithms. The performance of our models was assessed using both sweep frequency and fixed frequency radio burst data. The incorporation of an expanded feature space, which encompasses additional CME and radio burst features, likely contributed to the enhanced predictive capability of our model. Additionally, the limitation to $\ge$M2.0 class flares resulted in a more balanced dataset, which further improved the model’s performance. Our findings underscore the importance of reliable insights into the relationships between solar phenomena and >10 MeV SEP events. Features such as CME characteristics (width, linear speed), flare characteristics (intensity, integral soft X-ray), and radio burst frequencies (types II) were identified as crucial predictors. To ensure model generalization and mitigate overfitting, our methodology incorporated nested cross-validation, enabling robust evaluation of model performance and insights into model reaction variance in perturbed data. While our model demonstrates significant advancements in space weather forecasting, it is essential to acknowledge its limitations. Our model focuses specifically on predicting SEP events associated with strong flares, with a criterion of predicting SEP events associated with flares greater than M2.0. Thus, it may not capture SEP events associated with weaker flares. Additionally, our model excludes any event with missing values for its parameters, as complete data is required for accurate predictions. Our model is limited to cases where all types of phenomena occur (solar flare, CME and radio bursts) and the corresponding information is available in order to make a prediction.

## Methods

To anticipate the occurrence of SEP events, we must first characterize the phenomena that are used in the prediction.

- SEPs are protons, electrons, and heavier nuclei such as He-Fe undergoing acceleration from a few keV to GeV energies in at least two distinct regions: namely, solar flares and CME-driven interplanetary shocks. SEPs accelerated during flares are recognized as impulsive SEP events, while particle populations accelerated by near-Sun CME-shocks are denoted as gradual SEP events. Those associated with CME shocks observed near the Earth are termed energetic storm particle (ESP) events^2^.
- A solar flare is a transient brightening of the entire electromagnetic spectrum most commonly caused by magnetic reconnection in the corona of the Sun. To identify solar flares, the peak emission in SXR observed by the 1–8 Å channel of the GOES X-ray sensor (XRS) devices is employed. Class X flares have the largest peak in SXR emission, followed by classes M, C, B, and A^2^.
- A CME is a massive plasma outburst from the solar corona. Ejecta typically travels with a speed of a few hundred kilometres per second, while CMEs have been detected at speeds surpassing 2,000 kilometres per second in big occurrences. CMEs, particularly fast ones, have been shown to occur in tight spatial-temporal association with solar flares on numerous occasions.
- Radio bursts: The study of solar radio bursts opens new avenues for the investigation of significant precursors in solar physics. It is critical to distinguish between fixed and sweep frequencies radio bursts. The first type only analyses discrete frequencies and measures microwave peak values at these frequencies. There are several varieties of sweep-frequency radio bursts that can be recognised based on their shape. In the 1960s, solar radio bursts with frequencies less than a few hundred MHz were divided into five types^16^. Table 1 includes solar radio burst classifications with its characteristics.

SEPs and solar radio bursts are phenomena closely linked to solar flares and CMEs. Type III solar radio bursts often accompany the acceleration of electrons during solar flares, contributing to SEP generation. Conversely,Type II radio bursts are associated with shock wave propagation driven by CMEs. These bursts are produced when shock-accelerated electrons excite Langmuir waves at the local plasma frequency. The observed radio emissions result from nonlinear interactions between Langmuir waves and other plasma waves, such as ion-acoustic waves. These interactions convert energy into electromagnetic waves at the plasma frequency or its harmonic^17^. Type IV bursts are indicative of prolonged particle acceleration during CMEs and are linked to significant SEP production. By leveraging the timing and characteristics of solar radio bursts, we aim to forecast the occurrence of SEP events.Protons are the most abundant component of SEPs, which has a significant impact on both space and Earth. SEP occurrences in space can cause cancer in astronauts and cause the destruction of electronic components in spaceships. Solar cell degradation is related to SEP flux intensities at lower energies, whereas nuclear interactions are associated with SEP flux intensities at higher energies, such as sensor background noise, ionization, and displacement damage. SEPs on Earth can potentially irradiate passengers and flight crew in aeroplanes travelling at polar latitudes^2^. Forecasting SEP events improves the mitigation of adverse effects. Predicting SEP occurrences is challenging, not only because they involve complex physical processes still under study but also due to their rarity.

Our research employs an integrated approach that incorporates solar flare, CME, and radio burst data to anticipate SEP incidents. This task is framed as a supervised classification assignment, wherein solar phenomena are categorized as either zero or one: zero indicating the absence of a correlated SEP occurrence, and one signifying the presence of such an event.In this section, the data and methods that were used in our study are described.

### Dataset

We obtained data from three sources to predict the occurrence of SEP events:

- Solar flare and radio burst data were acquired from NOAA/SWPC. The SWPC Solar Event List serves as a comprehensive repository containing both observed and predicted solar activities, including solar flares, CMEs, and other relevant phenomena^18^. Entries in the SWPC Solar Event List typically include details such as flare classification (e.g., X, M, or C), time of occurrence, associated solar active region, and potential impacts on Earth’s space environment. In addition, information on detected CMEs, including estimated speed, propagation direction, and potential effects on Earth’s magnetosphere, as well as details on radio bursts and their intensity, may be available. To compile a comprehensive training dataset, we utilize the FTP link to download solar phenomena-related data spanning from 1997 to 2022. In the context of solar flares, parameters such as flare peak, logarithm of flare peak, flare intensity, rise time, duration, heliolongitude, heliolatitude, and integral SXR (a measure of intensity multiplied by duration) are considered. For sweep frequency radio bursts, parameters including rise time, duration, intensity, frequency range, and integral are analyzed for type II and III bursts. For fixed frequency radio bursts at 245, 410, 606, 1415, 2695, 4995, 8800, and 15,400 MHz, parameters such as rise time, duration, intensity, and integral are examined.
- The SEP data are acquired from the NOAA/NASA (National Aeronautics and Space Administration) SEP list^19^, which acts as a centralized repository of documented solar energetic particle occurrences. This compilation, collaboratively curated by NOAA/NASA, offers crucial insights into the intensity of SEP events and associated phenomena, such as solar flares and coronal mass ejections. The dataset covers observations from 1976 to 2022.
- CMEs data is acquired from LASCO observations conducted by the Solar and Heliospheric Observatory (SOHO) between 1997 and 2022, which are employed in our study. These identifications are integrated into the Coordinated Data Analysis Web (CDAW) online CME Catalog^20^. In our analysis, parameters such as angular width, linear speed, second order speed initial, second order speed final, and second order speed at 20 R$_{sun}$ are utilized.

### Data pre-processing

Upon downloading the data files, we began by performing pre-processing tasks. The analysis was conducted using the open-source Python programming language (https://www.python.org/) and its associated modules. For scientific data management and analysis, we employed the Python libraries NumPy (https://numpy.org/) and pandas (https://pandas.pydata.org/). To implement machine learning models and perform cross-validation, we utilized classification methods from the Python machine learning library, scikit-learn (https://scikit-learn.org/stable/). Between 1997 and 2022, we handled 115,424 flare events within the solar flare and radio burst records. These data are compiled into a file named ’SWPC’ during the initial preprocessing phase. Subsequently, we analyze the second NASA/NOAA file containing SEP data, detailing SEP occurrences and associated events such as solar flares and CMEs. This file documents 135 events spanning from 1997 to 2022, of which 106 are kept for further analysis after preprocessing. As part of the pre-processing procedure, SEP events that lack association with solar flare peaks or solar flare locations are excluded. The data extracted from the NASA/NOAA file include the date of the SEP event, the flux of the SEP, and details regarding the associated flare, such as the date, peak, and location of the flare. The resulting file is saved under the name “SEP”. Finally, we access the CME data files and compile all CME information from November 1997 to April 2022, containing a total list of 31,661 CME events. The data extracted from the CME files include the angular width of the CME, linear speed, as well as the second order speed initial, second order speed final, and second order speed at 20 R$_{sun}$. This collected information is then stored in a file named ’CME’.

We generate two data sets from the SWPC file. The first data set consists of flares and their associated sweep-frequency radio bursts . This data set contains the following information: the X-ray flare peak, the event ID, start, max, and end of SXR flux, location, as well as the intensity, frequency range, rise time, and duration of each type of radio burst. This information is saved in a file called the’sweep frequency dataset’. The second data set contains flares with radio bursts that have a definite frequency like 245, 410, and 8800 MHZ. This dataset, which is saved in a file with the name ’fixed frequency dataset’, includes the X-ray flare peak and event day, event ID, Start, Max, and End of the X-ray flare, location of the flare and radio burst at these frequencies (245, 410, 606, 1415, 2695, 8800, and 15400 MHZ), rise time of each frequency, intensity of each frequency, and duration.

After completing the cleanup of the SEP file, we utilize this data as Boolean information in both the sweep and fixed frequencies radio burst files. We scan both the sweep and fixed frequencies files for flare events occurring within the preceding 24 h of the SEP occurrence if it exists. Once identified, we compare the peak of the flare to the corresponding event’s flare peak value associated with SEP. In cases where multiple events share the same peak within the last 24 h, we locate the flare’s coordinates and select the one closest to them. Following this procedure, 104 events from the SEP file used as Boolean information in the two files (sweep and fixed). However, two SEP events that occurred on November 4, 2003, and January 2, 2016, were ignored because there was no corresponding flare peak in the two files equal to the associated flare peak with SEP events. The two files denote this information as a Boolean property, where the true class (‘a SEP event is expected’) signifies that the solar event caused a SEP event, while the false class (‘no SEP event is expected’) indicates the absence of a SEP event associated with the solar event.

Afterwards, the data undergoes a two-step filtering process: Initially, any flare event in the two files that is not associated with a SEP event and not linked to any type of radio burst is disregarded. Subsequently, events below the M2 level are also excluded. In fact, we have extracted 135 SEP events during the period of 1997-2022. After cleaning the data to remove events with missing values, we retained 106 SEP events. We found that 22 (approximately 20%) of these SEP events were associated with C and M1 class flares, with 11 events for each class.Also, negative class events are more common in the smaller intensity levels, so to focus on the most impactful events, we excluded all events below the M2.0 class flares, both positive and negative. This approach aligns with^4^, who concentrated on higher intensity flares to enhance the robustness and applicability of the findings, given that these stronger events are more likely to have severe effects on humans and infrastructure

After applying the filter, we extracted 639 solar flare events from the sweep frequency file, 945 solar flare events from the fixed frequency file, and 81 events from the SEP file. We excluded one event from the SEP file due to the presence of two events with the same associated flare occurring on March 28, 2022, sharing identical peak and location. These events happened on both March 28, 2022, and March 31, 2022. Consequently, we excluded the event from March 31, 2022, resulting in a total of 80 events. These events were labelled as true/false in the two files: 80 events labelled as positive and 558 as negative in the sweep frequency file, and 80 labelled as positive and 864 as negative in the fixed frequency file.

A real-time SEP occurrence prediction model should typically make its forecasts before the start of the SEP occurrences. While we have checked the availability of radio burst information prior to SEP onset, it is also crucial to consider the availability of CME data. For the SOHO/LASCO data used in this analysis, there is generally a  6 hour downtime^9^. This delay must be considered when discussing the feasibility and accuracy of real-time forecasting models.Then we check two data files that are accessible before the start time of the SEP events and may be used to train such models. Table 2 illustrates the difference between the beginning of the SEP event and the end of the radio burst at fixed frequencies. The event that occurred on November 22, 2001, was not associated with fixed-frequency radio bursts. In Table 3, it is observed that some of the end times of radio bursts at fixed frequencies are anticipated to occur after the beginning of the SEP event, but the probability of this occurrence is low. When calculating the average warning time, we use the most delayed precursor time in general. If the delay time of fixed frequency events is negative, this time is excluded from the calculation.

For the data integration, we merged the data from the two files (sweep and fixed frequency) with the CME file, using the date of flare occurrence as the primary linking parameter. We then performed the SF-CME association based on both temporal proximity^21,22^ and spatial correlation^23^, as detailed in^24^.To ensure the robustness of our flare-CME associations, we validated the results using the Database Of Notifications, Knowledge, Information (DONKI)^25^, which provides manually curated flare-CME links. Additionally, we cross-referenced our associations with the SF-CME association catalog provided by^9^. This multi-step validation process confirmed the reliability of our data integration and association methodology. After completing the association process, we identified 534 solar flare events associated with CMEs in the sweep frequency file and 740 events in the fixed frequency file. Then before creating the prediction model, categorical columns are converted to numerical columns. We start by changing the ‘location’ column to the heliolongitude and heliolatitude columns. The heliolatitude can take an integer value between -90 (S90, i.e., south 90) and +90 (N90, i.e., north 90), while the heliolongitude can take values below 90 (W90, i.e., west 90) and above -90 (E90, i.e., east 90). Second, we save the flare peak as classes C, M, and X, which are $10^{-6}$, $10^{-5}$, and $10^{-4}$ Watts/$m^{2}$, respectively, and create a new column called flare log, which is the logarithm of the flare peak values. Finally, we determined each phenomenon’s rising time, duration, and Integral SXR. The intensity of the solar flare and the duration of the phenomenon in minutes were simply multiplied to determine these approximative integrals. In the sweep frequency file, we also incorporated two additional columns. The first column labelled “*product_II*,” represents the multiplication of the integral of SXR with the integral of Type II. The second column, labelled “*product_III*,” represents the multiplication of the integral of SXR with the integral of Type III^4^. The input parameters for the model in the sweep frequency file encompass various aspects of solar flare and radio burst characteristics. These include the rise time of the flare, defined as the difference between its beginning and maximum times; the duration of the flare time, calculated as the difference between its beginning and end times; Flare Intensity is quantified by the flare peak flux measured in $( \text {cm}^{-2} \cdot \text {s}^{-1})$ which is represent of the number of X-ray photons detected per square centimeter per second; Flare Peak, expressed in $(\text {W} \text {m}^{-2})$; the logarithm of flare peak (*LOG_Flare*); heliolongitude; heliolatitude; and the integral of SXR, determined by multiplying the flare’s intensity by its duration. For CMEs parameters, we include angular width, linear speed, second-order speed initial, second-order speed final, and second-order speed at 20 R$_{sun}$). In addition, parameters related to radio bursts are incorporated, such as the rise time,which is labeled as ’RS_’ and computed as the difference between the time of maximum intensity and the initial onset of the radio burst; the duration of the radio burst, which is labeled as ’DUR_’ and obtained as the difference between its end and beginning time; radio burst intensity, categorized on a scale from 1 (low) to 3 (high); start frequency, which is labeled as ’FRQ_start_’; end frequency, which is labeled as ’FRQ_end_’; integral of the radio burst which is labeled as ’Integral_’, derived from multiplying its intensity by duration; and the product of the integral with respect to radio bursts, representing the product of SXR and radio burst integrals. These parameters are also utilized in the fixed frequency file; however, the fixed frequency file utilizes the the existence of these frequencies, which are labeled as ’Frq_’ (at frequencies 245, 410, 606, 1415, 2695, 4995, 8800, 15,400),rise time, duration, integral, and intensity at frequencies (245, 410, 606, 1415, 2695, 4995, 8800, 15,400) instead of Type II and III. To address missing values, we chose the median value for filling. This choice is preferable over the mean value, as the median is less influenced by outliers.

### ML model generation

The distribution of the data was skewed before the prediction model was created. This issue occurs when there are much fewer examples of the class of interest than there are of the other classes^26^. Three methods (sampling, cost-sensitive, and instance-weighting) are available to address this issue. Sampling techniques, including both over-sampling and under-sampling, are employed to mitigate class imbalance issues by altering the data distribution, ultimately leading to a more balanced class distribution. Cost-sensitive learning combines approaches at the algorithmic, data, or both levels, considering higher costs for misclassifying examples of the positive class in comparison to the negative class and attempting to minimize higher-cost errors^27^. The weight assigned to each instance in the data so that each class has the same overall weight and the sum of all weights across all instances is known as the instance weight.

In our study, we implemented nested cross-validation on the original dataset, utilizing five folds in both the inner and outer loops, for various models including decision tree (dtree), random forest (RF), support vector machine (svm), and linear support vector machine (linsvm). We utilized the code (SolarML/SEP-ML 2021)^28^ from the Lavasa model but made some modifications to the weights and applied only four models (RF, dtree, svm, linsvm). To optimize models performance, we employed randomized search, prioritizing the attainment of the highest F1-Score. In addition, we integrated stratified cross-validation and introduced weights to ensure equitable representation across different class distributions. Nested Cross-Validation (Nested CV) stands as a robust technique for estimating model performance while minimizing bias in model selection. Nested cross-validation operates through two primary loops: the outer and inner loops. In the Outer Loop, the dataset is partitioned into five folds. Each fold is sequentially treated as a validation set, while the remaining folds are leveraged for training. In the Inner Loop, 5-fold cross-validation is conducted within each training set of the outer loop. The cross-validation is employed for the model selection, encompassing hyperparameter tuning, across the training folds. Within the process of tuning models in nested cross-validation, we employ randomized search. Randomized search serves as a hyperparameter optimization technique designed to efficiently explore the best set of hyperparameters for an ML model. Unlike grid search, which systematically explores all potential combinations of hyperparameters within a predefined grid, randomized search samples a fixed number of hyperparameter configurations from a specified distribution. Additionally, we applied stratified splits/data partitions in nested cross-validation, a technique that ensures the distribution of target classes remains consistent across the folds. This method helps prevent biases in model evaluation, particularly when dealing with imbalanced datasets where certain classes may be underrepresented. By preserving the class distribution during cross-validation, stratified sampling enhances the reliability of model performance estimates.

In our study, we employed four techniques to address the problem of class imbalance: under-sampling, oversampling, SMOTE, and ADYSAN. Among these techniques, SMOTE (Synthetic Minority Over-sampling Technique)^29^ emerged as the method yielding the best F1-score. Consequently, we adopted SMOTE as the primary approach to balance the dataset. SMOTE is a widely used oversampling method that generates synthetic instances of the minority class by interpolating between existing minority class samples^30^. This technique effectively augments the representation of the minority class, leading to a more balanced dataset.

We applied machine learning models on three cases:

1. Imbalanced data: The original dataset was used without any modifications, retaining its inherent class imbalance.
2. Balanced data: The Synthetic Minority Over-sampling Technique (SMOTE) was applied to the entire dataset, creating an even class distribution across both the training and test sets.
3. Hybrid method: SMOTE was applied exclusively to the training set. This approach addressed class imbalance during training while keeping the test set in its original, imbalanced state.

We assess model performance using probabilistic measures in binary classification, where outcomes include True Positive (TP), False Positive (FP), True Negative (TN), and False Negative (FN). Key metrics used in this paper are Probability of Detection (POD), False Alarm Rate (FAR), F1-Score, True Skill Score (TSS), and Heidke Skill Score (HSS), as defined in Lavasa et al^9^.

In feature importance analysis, we focus on the Random Forest model, which is known for its superior performance. In this model, feature importance is determined by analyzing a collection of decision trees. The importance of each feature is calculated based on its ability to reduce impurity or error (such as Gini impurity) when splitting the data. Features that contribute significantly to impurity reduction are given higher importance. In our study, we used the feature_importances_ attribute in the Random Forest model to measure the impact of each feature on the model’s predictive performance. Through this attribute, we were able to assess the relative importance of features based on their contribution to reducing impurity and increasing homogeneity within the trees (Supplementary Information).

## Supplementary Information

Supplementary Information.

## Data Availability

The data that supports the findings of this study is available at ftp://ftp.swpc.noaa.gov/pub/indices/ and https://cdaw.gsfc.nasa.gov/CME_list/

## References

[1] Forbush, S. E. Solar cosmic rays. *Phys. Rev.* **70**, 771 (1946).

[2] Malandraki, O. E. & Crosby, N. B. (eds.) *Solar Particle Radiation Storms Forecasting and Analysis* (Springer, 2018).

[3] Whitman, K. et al. Review of solar energetic particle models. In *Advances in Space Research* (2022).

[4] Núñez, M. & Paul-Pena, D. Predicting 10 mev SEP events from solar flare and radio burst data. *Universe* **6** (2020).

[5] Aminalragia-Giamini, S. et al. Solar energetic particle event occurrence prediction using solar flare soft x-ray measurements and machine learning. *J. Sp. Weather Sp. Clim.* **11**, 59 (2021).

[6] National Oceanic and Atmospheric Administration. NOAA Space Weather Scales (n.d.). 10.1097/00004032-200007000-00014 10855782

[7] Laurenza, M., Alberti, T. & Cliver, E. W. A short-term Esperta-based forecast tool for moderate-to-extreme solar proton events. *Astrophys. J.* **857**, 107 (2018).

[8] Stumpo, M. et al. Open issues in statistical forecasting of solar proton events: A machine learning perspective. *Sp. Weather* **19** (2021).

[9] Lavasa, E. et al. Assessing the predictability of solar energetic particles with the use of machine learning techniques. *Solar Phys.* **296**, 107 (2021).

[10] Kasapis, S. et al. Interpretable machine learning to forecast SEP events for solar cycle 23. *Sp. Weather* **20** (2022).

[11] Núñez, M. Evaluation of the UMASEP-10 version 2 tool for predicting all 10 mev SEP events of solar cycles 22, 23 and 24. *Universe* **8**, 35 (2022).

[12] Balch, C. C. Updated verification of the space weather prediction center’s solar energetic particle prediction model. *Sp. Weather* **6** (2008).

[13] Laurenza, M. et al. A technique for short-term warning of solar energetic particle events based on flare location flare size and evidence of particle escape. *Sp. Weather Int. J. Res. Appl.* **7**, 20 (2009).

[14] Reames, D. V. The two sources of solar energetic particles. *Sp. Sci. Rev.* **175**, 53–92. 10.1007/s11214-013-9958-9 (2013).

[15] Núñez, M. Predicting solar energetic proton events (e 10 mev). *Sp. Weather* **9** (2011).

[16] Wild, J. P. & Smerd, S. F. Radio bursts from the solar corona. *Annu. Rev. Astron. Astrophys.* **10**, 159–96 (1972).

[17] Mann, G., Jansen, S. & Aurass, R. Excitation of Langmuir waves at shocks and solar type II radio bursts. *Astron. Astrophys.* **661**, 1–10. 10.1051/0004-6361/202142201 (2022).

[18] NOAA/SWPC. SWPC solar event list. ftp://ftp.swpc.noaa.gov/pub/indices/events/.

[19] NOAA/NASA. NOAA/NASA SEP List. ftp://ftp.swpc.noaa.gov/pub/indices/SPE.txt.

[20] CDAW. Coordinated data analysis web (CDAW) CME catalog. https://cdaw.gsfc.nasa.gov/CME_list/.

[21] Vršnak, B., Magdalenic, J., Aurass, H. & Mann, G. Band-splitting of coronal and interplanetary type ii bursts. *Astron. Astrophys.* **426**, 1093–1101. 10.1051/0004-6361:20041055 (2004).

[22] Vršnak, B., Sudar, D. & Ruždjak, D. The CME-flare relationship: Are there really two types of CMES?. *Astron. Astrophys.* **435**, 1149–1157. 10.1051/0004-6361:20042416 (2005).

[23] Youssef, M. Solar flare and coronal mass ejection characteristics during solar cycle 23. *Solar Phys.* **281**, 411–426. 10.1007/s11207-012-0174-3 (2012).

[24] Papaioannou, A., Sandberg, I., Anastasiadis, A. et al. Solar flares, coronal mass ejections, and solar energetic particle event characteristics. *JSWSC* **6**, A42. 10.1051/swsc/2016035 (2016).

[25] NASA CCMC. Database of Notifications, Knowledge, Information (DONKI) (2024).

[26] López, V. et al. An insight into classification with imbalanced data: Empirical results and current trends on using data intrinsic characteristics. *Inf. Sci.* **250**, 113–141 (2013).

[27] Weiss, G. M., McCarthy, K. & Zabar, B. Cost-sensitive learning vs. sampling: Which is best for handling unbalanced classes with unequal error costs. *Dmin* **7**, 24 (2007).

[28] SolarML. SolarML/sep-ml (2021).

[29] Chawla, N. V., Bowyer, K. W., Hall, L. O. & Kegelmeyer, W. P. SMOTE: Synthetic minority over-sampling technique. *J. Artif. Intell. Res.* **16**, 321–357 (2002).

[30] Benella, S. et al. Statistical treatment of solar energetic particle forecasting through supervised learning approaches. In *Proceedings of Science (ECRS)* (2023).

