# Supplementary material for: Forecasting solar energetic particles using multi-source data from solar flares, CMEs, and radio bursts with machine learning approaches
Source: Sci Rep. 2025 Mar 19;15:9546. doi: 10.1038/s41598-025-92207-1 (PMC11923114; doi:10.1038/s41598-025-92207-1)
Supplement: Supplementary file 1 — Supplementary Information. [file 41598_2025_92207_MOESM1_ESM.pdf]

Appendix A: Hyper-Parameter Spaces

A. Decision Tree (dtree):

a. `[criterion:]` 'gini', 'entropy'

b. `[min_samples_leaf:]` range(start=2, end=100, step=2)

c. `[min_samples_split:]` range(start=5, end=130, step=5)

d. `[max_depth:]` 2, 4, 6, 8, 10, 20, 30, 40, 50, 60, 70, 80, 90, 100, None

e. `[class_weight:]` [0:x, 1:1.0-x for x in weights] where (weights = np.linspace(0.0,0.99,300))

B. Random Forest (RF):

a. `[criterion:]` 'gini', 'entropy'

b. `[n_estimators:]` 10, 20, 40, 60, 80, 100, 200, 400, 600, 800, 1000

c. `[min_samples_leaf:]` range(start=2, end=100, step=2)

d. `[min_samples_split:]` range(start=5, end=130, step=5)

e. `[max_depth:]` 2, 4, 6, 8, 10, 20, 30, 40, 50, 60, 70, 80, 90, 100, None

f. `[max_features:]` 'auto', 'sqrt', 'log2'

g. `[class_weight:]` [0:x, 1:1.0-x for x in weights] where (weights = np.linspace(0.0,0.99,300))

C. Support Vector Machines with Linear Kernel (linsvm):

a. `[penalty:]` 'l2', 'l1'

b. `[random_state:]` 42

c. `[C:]` logspace(start=-4, end=3, step=1)

d. `[max_iter:]` 5000, 10000, 20000

e. `[class_weight:]` [0:x, 1:1.0-x for x in weights] where (weights = np.linspace(0.0,0.99,300))

D. Support Vector Machines with Non-linear Kernel (svm):

a. `[kernel:]` 'rbf', 'poly', 'sigmoid'

b. `[C:]` logspace(start=-4, end=3, step=1)

c. `[gamma:]` 'scale', 'auto', 0.001, 0.01, 0.1, 1, 10

d. `[degree:]` 2, 3, 4

e. `[coef0:]` -10, -1, -0.1, -0.01, -0.001, 0.0, 0.001, 0.01, 0.1, 1, 10

f. `[max_iter:]` 5000, 10000, 20000

g. `[class_weight:]` [0:x, 1:1.0-x for x in weights] where (weights = np.linspace(0.0,0.99,300))

Appendix B: Results of Models

Imbalance case (sweep frequency)

- **Decision tree (dtree):**

- **Linear support vector machine (linsvm):**

- **Nonlinear Support vector machine (svm):**

Table S1. dtree performance on sweep frequency data (imbalance)

Folds	F1	POD	FAR	TSS	HSS	Min_split	Min_leaf	Max_depth	criterion	Class weight
Fold1	0.63	0.81	0.48	0.68	0.55	120	46	60	Entropy	{0: 0.3708, 1: 0.6291}
Fold2	0.71	0.69	0.27	0.64	0.66	30	18	50	Gini	{0: 0.2218, 1: 0.7782}
Fold3	0.75	0.75	0.25	0.71	0.71	50	5	None	Entropy	{0: 0.4205, 1: 0.5795}
Fold4	0.69	0.75	0.37	0.67	0.62	40	12	20	Entropy	{0: 0.4569, 1: 0.5431}
Fold5	0.65	0.81	0.46	0.69	0.57	35	7	4	Gini	{0: 0.2318, 1: 0.7682}
avg	0.68	0.76	0.36	0.67	0.62					
std	0.04	0.04	0.09	0.02	0.05					

Table S2. linsvm performance in sweep frequency data (imbalance)

Folds	F1	POD	FAR	TSS	HSS	Penalty	C	Max_iter	Class weight
Fold1	0.72	0.81	0.35	0.74	0.67	L2	0.1	10000	{0: 0.2947, 1: 0.7053}
Fold2	0.72	0.81	0.35	0.74	0.67	L2	100	20000	{0: 0.2185, 1: 0.7815}
Fold3	0.65	0.81	0.46	0.69	0.57	L2	10	20000	{0: 0.1755, 1: 0.8245}
Fold4	0.75	0.94	0.38	0.84	0.7	L2	1	20000	{0: 0.2914, 1: 0.7086}
Fold5	0.58	0.56	0.4	0.5	0.51	L2	0.1	20000	{0: 0.3675, 1: 0.6325}
avg	0.68	0.78	0.38	0.70	0.62				
std	0.06	0.12	0.04	0.11	0.07				

- **Random forest (RF):**

Imbalance case (fixed frequency)

- **Decision tree (dtree):**

- **Linear support vector machine (linsvm):**

Table S3. svm performance on sweep frequency data (imbalance)

folds	F1	POD	FAR	TSS	HSS	Max_iter	Kernal	gamma	degree	Coeff	C	Class weight
Fold1	0.69	0.75	0.37	0.67	0.62	5000	rbf	scale	4	10	1	{0:0.3543,1:0.6457}
Fold2	0.71	0.62	0.17	0.6	0.67	10000	rbf	0.1	3	10	1000	{0:0.2616,1:0.7384}
Fold3	0.7	0.81	0.38	0.72	0.64	20000	rbf	0.1	4	-1	1000	{0:0.0199,1:0.9801}
Fold4	0.72	0.88	0.39	0.78	0.66	10000	rbf	0.1	3	0.1	1000	{0:0.0430,1:0.9570}
Fold5	0.69	0.75	0.37	0.67	0.62	10000	rbf	0.1	2	0.001	1	{0:0.3410,1:0.6590}
avg	0.70	0.76	0.33	0.68	0.64							
std	0.01	0.08	0.08	0.05	0.02							

Table S4. RF performance on sweep frequency dataset (imbalance)

folds	F1	POD	FAR	TSS	HSS	n_ estimator	Min_ split	Min_ leaf	Max_ feature	Max_ depth	criterion	Class weight
Fold1	0.72	0.81	0.35	0.74	0.67	40	65	28	log2	70	Entropy	{0: 0.2781,1: 0.7218}
Fold2	0.77	0.75	0.2	0.72	0.74	60	5	4	sqrt	4	Entropy	{0:0.18541,1:0.8146}
Fold3	0.78	0.88	0.3	0.81	0.73	400	65	66	log2	20	Gini	{0:0.2351,1:0.7649}
Fold4	0.8	1	0.33	0.91	0.76	40	25	42	log2	None	Gini	{0:0.18541,1:0.814581}
Fold5	0.72	0.81	0.35	0.73	0.67	60	65	72	sqrt	4	Entropy	{0:0.2582,1:0.7418}
avg	0.75	0.85	0.30	0.78	0.71							
std	0.03	0.08	0.05	0.07	0.03							

- **Nonlinear support vector machine (svm):**

- **Random Forest (RF):**

Table S5. dtree performance on fixed frequency data (imbalance)

folds	F1	POD	FAR	TSS	HSS	Min_split	Min_ leaf	Max_ depth	criterion	Class weight
Fold1	0.65	0.69	0.39	0.63	0.6	65	8	4	Entropy	{0: 0.3145, 1: 0.6855}
Fold2	0.73	0.69	0.21	0.66	0.7	90	24	20	Entropy	{0:0.2483,1:0.7517}
Fold3	0.76	0.88	0.33	0.82	0.72	80	27	4	Entropy	{0:0.1688,1:0.8312}
Fold4	0.67	0.75	0.4	0.69	0.62	70	35	2	Entropy	{0:0.2815,1:0.7185}
Fold5	0.52	0.5	0.47	0.45	0.46	45	23	10	Entropy	{0:0.4304,1:0.5696}
avg	0.66	0.70	0.36	0.65	0.61					
std	0.08	0.12	0.08	0.11	0.09					

Table S6. linsvm performance on fixed frequency data (imbalance)

folds	F1	POD	FAR	TSS	HSS	Penalty	C	Max_iter	Class weight
Fold1	0.73	0.75	0.29	0.71	0.69	L2	0.01	20000	{0: 0.1622, 1: 0.8378}
Fold2	0.76	0.81	0.28	0.77	0.73	L2	0.01	10000	{0:0.1423,1:0.8577}
Fold3	0.62	0.88	0.52	0.76	0.56	L2	0.1	10000	{0:0.2350,1:0.7650}
Fold4	0.77	0.94	0.35	0.88	0.74	L2	0.01	20000	{0:0.1622,1:0.8378}
Fold5	0.65	0.88	0.48	0.78	0.6	L2	0.01	20000	{0:0.2019,1:0.7981}
avg	0.70	0.85	0.38	0.78	0.66				
std	0.06	0.06	0.09	0.05	0.07				

Table S7. svm performance on fixed frequency data (imbalance)

folds	F1	POD	FAR	TSS	HSS	Max_iter	Kernal	gamma	degree	Coeff	C	Class weight
Fold1	0.76	0.81	0.28	0.77	0.73	10000	rbf	auto	2	-0.001	10	{0:0.3112,1:0.6888}
Fold2	0.71	0.69	0.27	0.66	0.68	10000	rbf	auto	2	-0.001	10	{0:0.3112,1:0.6888}
Fold3	0.64	0.88	0.5	0.77	0.58	5000	rbf	auto	4	0.1	10	{0:0.3476,1:0.6524}
Fold4	0.74	0.88	0.36	0.81	0.7	20000	rbf	scale	2	-10	10	{0:0.3277,1:0.6723}
Fold5	0.65	0.88	0.48	0.78	0.6	10000	sigmoid	0.001	2	1	1000	{0:0.2582,1:0.7418}
avg	0.7	0.82	0.37	0.75	0.65							
std	0.04	0.07	0.09	0.05	0.05							

Table S8. RF performance on fixed frequency data (imbalance)

folds	F1	POD	FAR	TSS	HSS	n_ estimator	Min_ split	Min_ leaf	Max_ feature	Max_ depth	criterion	Class weight
Fold1	0.67	0.75	0.4	0.69	0.62	100	100	38	sqrt	10	Gini	{0: 0.2549, 1: 0.7451}
Fold2	0.76	0.69	0.15	0.67	0.73	100	60	24	sqrt	2	Entropy	{0:0.2516,1:0.7484}
Fold3	0.73	0.75	0.29	0.71	0.69	100	15	12	sqrt	80	Gini	{0:0.3079,1:0.6921}
Fold4	0.8	1	0.33	0.94	0.77	1000	105	34	sqrt	80	Gini	{0:0.2582,1:0.7418}
Fold5	0.62	0.62	0.38	0.58	0.58	100	65	20	sqrt	50	Entropy	{0:0.2947,1:0.7053}
avg	0.71	0.76	0.31	0.71	0.67							
std	0.06	0.12	0.08	0.11	0.06							
